# Soil enzyme responses to land use change in the tropical rainforest of the Colombian Amazon region

**DOI:** 10.1371/journal.pone.0255669

**Published:** 2021-08-18

**Authors:** Adriana M. Silva-Olaya, Dúber A. Mora-Motta, Maurício R. Cherubin, Daniel Grados, Anil Somenahally, Fausto A. Ortiz-Morea

**Affiliations:** 1 Amazonian Research Center CIMAZ-MACAGUAL, University of the Amazon, Florencia, Colombia; 2 Department of Soil Science, ‘‘Luiz de Queiroz” College of Agriculture, University of Sao Paulo, Sao Paulo, Brazil; 3 Instituto del Mar del Perú, Esquina Gamarra y General Valle s/n Chucuito, Callao, Perú; 4 Department of Soil and Crop Sciences, Texas A&M University, Overton, Texas, United States of America; Feroze Gandhi Degree College, INDIA

## Abstract

Soil enzymes mediate key processes and functions of the soils, such as organic matter decomposition and nutrient cycling in both natural and agricultural ecosystems. Here, we studied the activity of five extracellular soil enzymes involved in the C, N, and P-mineralizing process in both litter and surface soil layer of rainforest in the northwest region of the Colombian Amazon and the response of those soil enzymes to land use change. The experimental study design included six study sites for comparing long-term pasture systems to native forest and regeneration practices after pasture, within the main landscapes of the region, mountain and hill landscapes separately. Results showed considerable enzymatic activity in the litter layer of the forest, highlighting the vital role of this compartment in the nutrient cycling of low fertility soils from tropical regions. With the land use transition to pastures, changes in soil enzymatic activities were driven by the management of pastures, with SOC and N losses and reduced absolute activity of soil enzymes in long-term pastures under continuous grazing (25 years). However, the enzyme activities expressed per unit of SOC did not show changes in C and N-acquiring enzymes, suggesting a higher mineralization potential in pastures. Enzymatic stoichiometry analysis indicated a microbial P limitation that could lead to a high catabolic activity with a potential increase in the use of SOC by microbial communities in the search for P, thus affecting soil C sequestration, soil quality and the provision of soil-related ecosystem services.

## Introduction

The extracellular enzymes play important functions in the biogeochemical cycles, catalyzing the reactions involved in organic matter decomposition, becoming critical drivers of carbon (C) storage and the supply of nutrients to tropical forest ecosystems [1]. The activity of extracellular enzymes to degrade the litter layer is mostly regulated by soil microbial community, supplying energy and nutrients for microbial and plant growth [2]. Therefore, reducing in the quantity and quality of the litter after land use change from native forests to agricultural uses could alter soil enzyme activity and nutrient biogeocycling, including soil organic carbon sequestration [3].

Several studies revealed that soil enzyme production and activity are significantly impacted by land use change and agricultural practices [4–8]. However, the response of each soil enzyme is highly variable [8–11]. It was clear, to some extent, that soil enzyme responses are site specific depending on climate and soil conditions (e.g., nutrient availability), plant community type, and land management [8], with each factor impacting soil enzymes differently [5, 7]. Thus, understanding soil enzyme responses to land use changes in different soil conditions is critical for restoring soil health and increase land productivity.

In the Amazon region, intensive deforestation of native rainforest for expansion of livestock and agricultural activities has led to significant changes of soil C stocks, and degradation of soil physico-chemical properties in many regions [12–15]. Particularly, soil health degradation and soil C declines were substantial in pasture systems with low-fertility soils [14–16]. It is also well established that replacing native rainforest diminishes quantity and quality of organic matter input to the soil, the primary source of microbial resources (e.g., C, N, S, P), and, therefore, significantly shifting soil microbial communities [17–19]. Consequently, soil enzymatic activity could be affected, with implications on soil C storage and ecosystem functioning.

Extracellular enzyme dynamics in forest ecosystems and their responses after land use change, particularly for those farms situated in low-fertility soils is not clearly understood. Furthermore, there is a paucity of studies of soil enzyme activities in the litter layer, an essential component for that ecosystem functioning. Those ecosystems are experiencing rapid changes due to direct human disturbance, particularly in the Colombian portion of the Amazon region, which has become an important hotspot of deforestation of that biome [20]. In this region, very little is known about the effects of land use changes on enzyme activities, especially on the impact of the transition from natural forest to pastures for livestock production.

In this sense, we performed this study by leveraging several long-term field sites to evaluate the enzymatic activity in both litter and surface soil layer in the rainforest of the northwest Colombian Amazon region and the soil enzyme responses to land use change. The long-term study sites included native forests, pastures, and abandoned pastures under natural regeneration. Additionally, forest and pasture systems were compared separately within the mountain and hill landscapes. We also assessed soil C, N, and P contents to establish the relationships with soil enzymes. We hypothesized that the litter layer in tropical Amazon forests maintains high enzymatic activity responsible for C, N and P mineralization. This layer will be lost when the forest is replaced by pasture, but due to legacy effects maintaining higher mineralization potential and loss of organic matter. We also hypothesized that high N and P nutrient demand is a major driver for mineralization potential in pasture systems situated in low-fertile soil.

## Material and methods

### Study site and land uses

**Fig 1:**
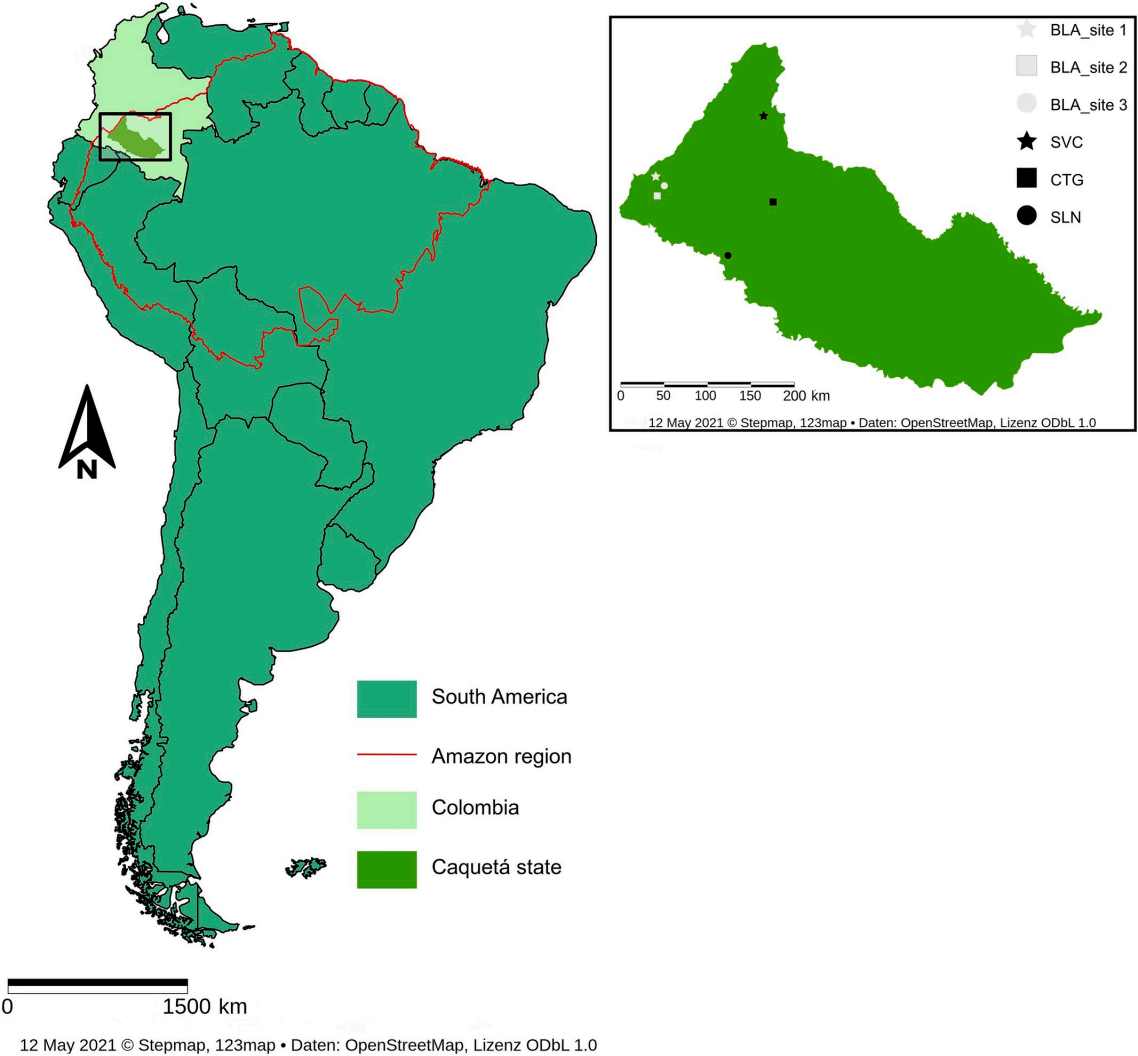
The geographic location of the study sites in the northwestern region of Colombian Amazon (BLA: Belén de los Andaquíes, SVC: San Vicente del Caguán, CTG: Cartagena del Chairá, SLN: Solano). Republished from StepMap.com under a CC BY license, with permission from StepMap GmbH, original copyright 2021.

The study was performed in the Caquetá state of northwestern Colombian Amazon, which has experienced vast deforestation of Amazon Basin, ranking second globally after the arc of deforestation in Brazil [21]. Six sites were strategically selected on the two main landscapes of the Colombian Amazon region: hills and mountain landscape [22], guaranteeing the representability of the study area (Fig 1). The three sites on the hills landscape were located in the municipalities of i) San Vicente del Caguán (SVC); ii) Cartagena del Chairá (CTG), and iii) Solano (SLN). Those sites are located within a range of altitude between 235 to 290 m.a.s.l. The three sites in the mountain landscape were located in the municipality of Belén de Los Andaquíes (BLA), in a region of Andes-Amazon transition with a maximum elevation of 1000 m.a.s.l. All selected sites respond to a significant rate of forest clearing for pasture establishments.

The regional climate is classified as a tropical rainforest—Af type (Koppen classification), with a mean annual temperature of 25.5°C and annual precipitation of 3793 mm. In the hills landscape, the soils are highly weathered, moderately deep, and classified as Typic Hapludults, which were originated from claystone. In contrast, the soils are less developed in the mountain landscape, classified as Lithic Udorthents originated from granite and gneiss with sandstone inclusions [22]. Both hill and mountain soils are very acid, with high Al saturation and low-cation exchange capacity and basic cation contents (Ca^2+^, Mg^2+^, K^+^ and Na^+^), resulting in poor-chemically soils [22].

A paired-site comparison involving a forest and adjacent pasture, which was established after forest slash and burning, was assessed in each site. Additionally, mountain landscape areas with natural regeneration after pasture abandonment (a typic transition in land use in the Colombian Amazon region) were also evaluated, totalizing six study areas in the hills landscape and nine study areas in the mountain landscape. The chemical characteristics and granulometric composition of the soils in all study areas are presented in Table 1.

**Table 1:** Soil granulometric fractions and chemical attributes in the study sites.

Landscape	Land use	Clay	Silt	Sand	pH	Ca	Mg	K	Al
		--------------g kg^-1^---------				----------------cmol kg^-1^ -----------------			
San Vicente del Caguán (SVC)						
**Hills**	**Forest**	292	246	462	4.19	4.94	3.36	0.22	2.52
	**Pasture**	261	267	472	3.94	0.40	0.24	0.12	2.16
		Solano (SLN)						
	**Forest**	319	320	361	4.10	0.18	0.15	0.44	4.40
	**Pasture**	331	260	409	4.08	0.49	0.21	0.31	2.35
		Cartagena del Chairá (CTG)						
	**Forest**	302	335	363	3,46	0.43	0.05	0.28	6.79
	**Pasture**	257	172	572	3.89	1.00	0.04	0.15	2.76
**Mountain**		Belén de los Andaquíes (BLA_site 1)						
	**Forest**	92	38	869	3.80	0.40	0.09	0.16	0.49
	**Pasture**	338	125	537	4.05	0.59	0.27	0.36	1.10
	**Nat. Regeneration**	228	120	651	3.83	0.96	0.26	0.30	1.99
		Belén de los Andaquíes (BLA_site 2)						
	**Forest**	251	118	631	3.84	0.48	0.18	0.34	1.20
	**Pasture**	209	133	658	4.62	0.70	0.26	0.60	0.60
	**Nat. Regeneration**	230	110	693	3.92	0.40	0.27	0.47	1.44
		Belén de los Andaquíes (BLA_site 3)						
	**Forest**	281.28	120.53	598.19	3.79	0.21	0.14	0.23	1.47
	**Pasture**	262.57	67.43	670.00	3.99	0.55	0.24	0.37	1.34
	**Nat. Regeneration**	264.45	83.36	652.19	3.83	1.00	0.19	0.19	1.42

The forest areas correspond to a typical Amazon rainforest, constituted by a vegetation community dominated by regularly distributed arboreal elements representing approximately 80% of the total area, forming a discontinuous canopy with a height greater than 15 meters. The pasture areas correspond to pastures composed of *Brachiaria sp*. established >25 years ago after slash and burn of native forests in hills landscape and 15 years ago in mountain landscape; with an occupation of approximately one cattle head by hectare. The natural regeneration areas correspond to 10-year-old secondary vegetation, established over abandoned pastures. In that region, it is common, after the pasture yield decreases because of the absence of investments in soil and pasture management practices (e.g., fertilization, liming, control of soil erosion and weeds, and soil compaction management) the landowners abandon the pastures allowing secondary succession.

### Soil sampling and analysis

In each area, soil sampling was done in four plots of 4 m^2^ spaced 100 m apart following a completely randomized design. Since most of the biochemical transformations occur in the topsoil layer, we collected ten soil cores (5-cm diameter x 10-cm depth) in 0–10 cm soil layer in each sampling plot. For that aim, all litter material was removed. Then all the individual samples were mixed to conform one composite sample per plot, which was placed in a sterile plastic bag, sealed, and transported (<24 h) under refrigeration (4°C) to the Biogeochemical Process Laboratory at the University of the Amazon (Florencia-Caquetá, Colombia).

In order to study the potential enzymatic activity of the litter layer, we also collected litter samples in the forest sites. For that, four quadrants of 25 x 25 cm were positioned around each soil sampling point, and all the litter inside it removed, weighed, and mixed into a composite sample, which was transported under refrigeration (4°C) to the laboratory. Subsequently, the litter samples at field-moist conditions were passed through a 2-mm sieve and all litter fraction lower than 2 mm was submitted to further analysis. No specific permits were required to collect soil and litter samples from the field locations, which were in non-protected land. The biochemical characterization of litter samples is presented in Table 2.

**Table 2:** Fresh matter and biochemical characteristics of forest litter layer from Colombian Amazon region.

Landscape	Study site	Total Fresh matter (Mg ha)	Biochemical characteristics (fraction < 2 mm)							
			C (%)	N (%)	P (%)	Hemicellulose (%)	Cellulose (%)	Lignin (%)	C:N	C:P	
**Hills**	**SVC**	24.70	23.59	1.27	0.13	19.20	15.09	11.81	18.82	185.05	
	**SLN**	22.50	20.59	1.06	0.05	14.89	21.27	11.08	16.65	433.68	
	**CTG**	21.50	26.01	1.42	0.10	18.80	18.34	10.49	18.22	266.79	
**Mountain**	**BLA_site 1**	23.15	23.96	1.20	0.06	24.09	20.7	12.57	19.99	416.81	
	**BLA_site 2**	30.40	24.16	1.23	0.09	9.86	16.21	11.96	19.60	259.10	
	**BLA_site 3**	31.91	26.55	1.29	0.12	10.84	15.22	11.56	20.50	225.96	

Soil biochemical attributes such as phosphorus (P), nitrogen (N), and soil organic carbon (SOC) contents were quantified. For that aim, a portion of soil samples collected was air-dried and sieved to 2 mm. Then, soil P content was measured by extraction with the Bray II method [23] and determined the P-molybdate blue color on a visible spectrophotometer at 660 nm. For soil organic C and N quantification, samples were ground to a fine powder and sieved to 150 μm before determination by dry combustion [24] using a CN 802 carbon nitrogen elemental analyzer (furnace at 1000°C in pure oxygen).

The absolute activity of five extracellular hydrolytic enzymes involving P, N and C-mineralizing processes in soil and forest litter samples was assayed using standard fluorometric microplate methods [25]. The activity of acid phosphatase (P mineralization), β-1,4-N-acetylglucosaminidase (chitin degradation), β-1,4-glucosidase (sugar degradation), β-D-cellobiohydrolase (cellulose degradation), β-D-xylosidase (hemicellulose degradation) was examined as described by [25].

Briefly, soil or forest litter slurries were prepared in a sodium acetate buffer with a pH closely with the soil pH. Then, 800 μl of soil slurry was pipetted into 96-well deep plates. Separate plates were prepared for 4-methylumbelliferone standard curves for each sample. A dose of 200 μl of appropriate standards and substrates were added to the soil slurries. Samples and standards were incubated for 3 h at room temperature. Then supernatants were pipetted into black 96-well plates and the fluorescence was measured at 365 nm excitation wavelength and 450 nm emission wavelength in the VarioSkan Lux multimode microplate reader. For minimizing "well-to-well variation" [25], three assay replicates in each plate were prepared. The absolute activities of each type of enzyme were expressed in units of nmol of product per gram of oven-dry weight soil/litter per hour.

For each soil land use, the geometric mean of soil enzyme activities (*GMea*) was calculated as:

$$GMea=\sqrt[{5}]{P+N+C\;cycling\;enzymes}$$

Where P denotes acid phosphatase, N corresponds to β-1,4-N-acetylglucosaminidase and C cycling enzymes denote the activity of β-1,4-glucosidase + β-D-cellobiohydrolase + β-D-xylosidase, respectively [26, 27].

Since absolute enzyme activity gives an estimate of the rate at which the product of activity is made available in the soil, we further calculated the specific enzyme activities (μmol mg C^−1^ h^−1^) by dividing the absolute enzyme activity (nmol g^−1^ soil h^−1^) by the soil total C (mg kg^−1^). The specific enzyme activity allows reliable comparison of soil with different land-use types and can give an insight into the nutritional status of the organic matter regarding soil microorganisms [5, 7]. Microbial C:N, C:P and N:P acquisition (*E*_*CN*_ *E*_*CP*_ and *E*_*NP*_, respectively) were calculated as:

$$E_{CN}=\ln\;C\;cycling\;enzymes/\ln\;N\;cycling\;enzyme\;$$

$$E_{CP}=\ln\;C\;cycling\;enzymes/\ln\;P\;cycling\;enzyme\;$$

$$E_{NP}=\ln\;N\;cycling\;enzyme/\ln\;P\;cycling\;enzyme$$

Where *C cycling enzymes* denote the absolute activity of β-1,4-glucosidase + β-D-cellobiohydrolase + β-D-xylosidase, *N cycling enzyme* corresponds to the absolute activity of β-1,4-N-acetylglucosaminidase and *P cycling enzyme* denotes the absolute activity of acid phosphatase, respectively.

### Statistical analyses

Changes in the variables assessed between forest litter and soil, as well as between land uses (forest and pastures), were analyzed using the Student’s Test (*p* <0.05 and *p* <0.01). In the mountain landscape, the effect of land use on the different variables was studied by adjusting a linear mixed effect model (lmer), which considered the land-use as a fixed factor and plots as random factors. The assumptions of normality and homogeneity of variance were evaluated using an exploratory residual analysis. When significant, the means of the variables were compared according to Tukey’s test (*p* <0.05). All analyses were conducted in statistical software R version 4.0.3 [28], using integrated development environment RStudio version 1.3.1. [29].

## Results

### Comparison of SOC, N and P content in the soil layer of different land use practices

Land use change from forest to pasture induced a decline in soil C and N content in the hills landscape at SVC and CTG sites (Table 3). In mountain landscape, the transition zones within forest, pasture and natural regeneration did not significantly alter SOC and N content at BLA_site 2 and site 3. Whereas significantly higher SOC and N content were observed in pasture and natural regeneration sites than forest sites at the BLA_site 1. Soil C:N ratio did not significantly differ among land use systems in both types of landscapes.

**Table 3:** Soil C, N, C:N ratio, and P in forest, pasture, and natural regeneration in the Colombian Amazon region.

Landscape	Land use	San Vicente del Caguán (SVC)			
Hills		C (g kg^-1^)	N (g kg^-1^)	C:N	P (mg kg^-1^)
	Forest	24.76 (2.01) a*	2.76 (0.23) a	9.03 (0.41) a	1.24 (0.15) a
	Pasture	12.05 (2.71) b	1.46 (0.17) b	8.72 (1.76) a	1.25 (0.11) a
		Cartagena del Chairá (CTG)			
	Forest	30.50 (1.63) a	3.12 (0.13) a	9.82 (0.52) a	2.24 (0.27) a
	Pasture	21.38 (2.32) b	2.31 (0.23) b	9.22 (0.15) a	1.65 (0.26) a
		Solano (SLN)			
	Forest	26.30 (1.94) a	2.53 (0.15) a	10.36 (0.15) a	3.25 (0.58) b
	Pasture	21.74 (0.99) a	2.17 (0.13) a	10.09 (0.45) a	5.51 (0.36) a
Mountain		Belén de los Andaquíes (BLA_site 1)			
	Forest	13.95 (1.57) b	1.30 (0.18) b	10.98 (0.38) a	8.93 (0.83) a
	Pasture	29.75 (1.28) a	2.80 (0.10) a	10.61 (0.15) a	3.17 (0.74) b
	Natural Regeneration	25.96 (3.07) a	2.88+0.32 a	8.98 (0.15) b	7.31 (0.81) a
		Belén de los Andaquíes (BLA_site 2)			
	Forest	35.08 (3.89) a	3.31 (0.33) a	10.59 (0.43) a	5.03 (0.99) ab
	Pasture	37.02 (3.75) a	3.56 (0.42) a	10.53 (0.31) a	3.17 (0.69) b
	Natural Regeneration	36.50 (2.36) a	4.44 (0.59) a	9.44 (0.90) a	8.36 (1.58) a
		Belén de los Andaquíes (BLA_site 3)			
	Forest	29.81 (2.47) a	3.27 (0.32) a	9.22 (0.42) a	4.71 (0.96) a
	Pasture	27.59 (2.87) a	2.67 (0.22) a	10.24 (0.37) a	3.26 (0.27) a
	Natural Regeneration	30.32 (3.37) a	2.90 (0.20) a	10.33 (0.50) a	2.90 (0.46) a

*Means and standard errors (in parenthesis) followed by the same lowercase letter in each study site did not differ among themselves according to the Student’s Test in hills landscape and Tukey’s HSD test and mountain landscape (*p* <0.05).

In general, low soil P contents were observed in all sites evaluated. The P content did not change due to pasture establishment in CTC and SVC sites in hills landscape as either in BLA _site 2 and site 3 of the mountain landscape. Subtle differences in P content between forest and pasture were only detected in SLN site and BLA_site 1 mountain landscape.

### Absolute enzyme activities in litter and soil layer of the tropical forest

**Fig 2:**
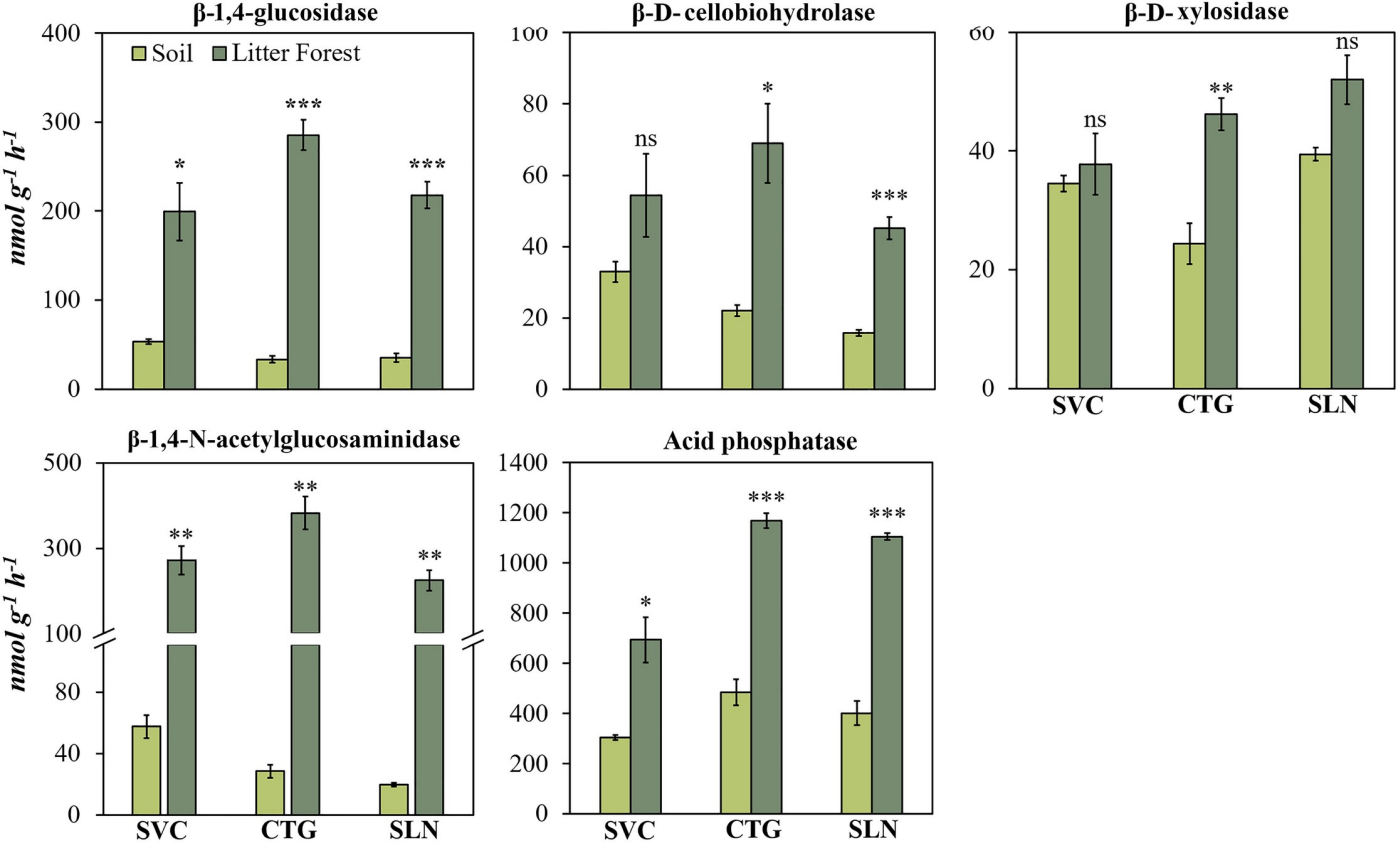
β-1,4-glucosidase, β-D-cellobiohydrolase, β-D-xylosidase, β-1,4-N-acetylglucosaminidase, acid phosphatase enzymes activity in soil and litter of the forest located in hill landscape sites (SVC: San Vicente del Caguan, CTG: Cartagena del Chaira, SLN: Solano) of Colombian Amazon region. Error bars denote standard error. Asterisks indicate that the means values differ significantly between themselves to the level *p* <0.05 (*), *p* <0.01 (**) and *p* <0.001(***) for each study site, according to the Student’s Test.

**Fig 3:**
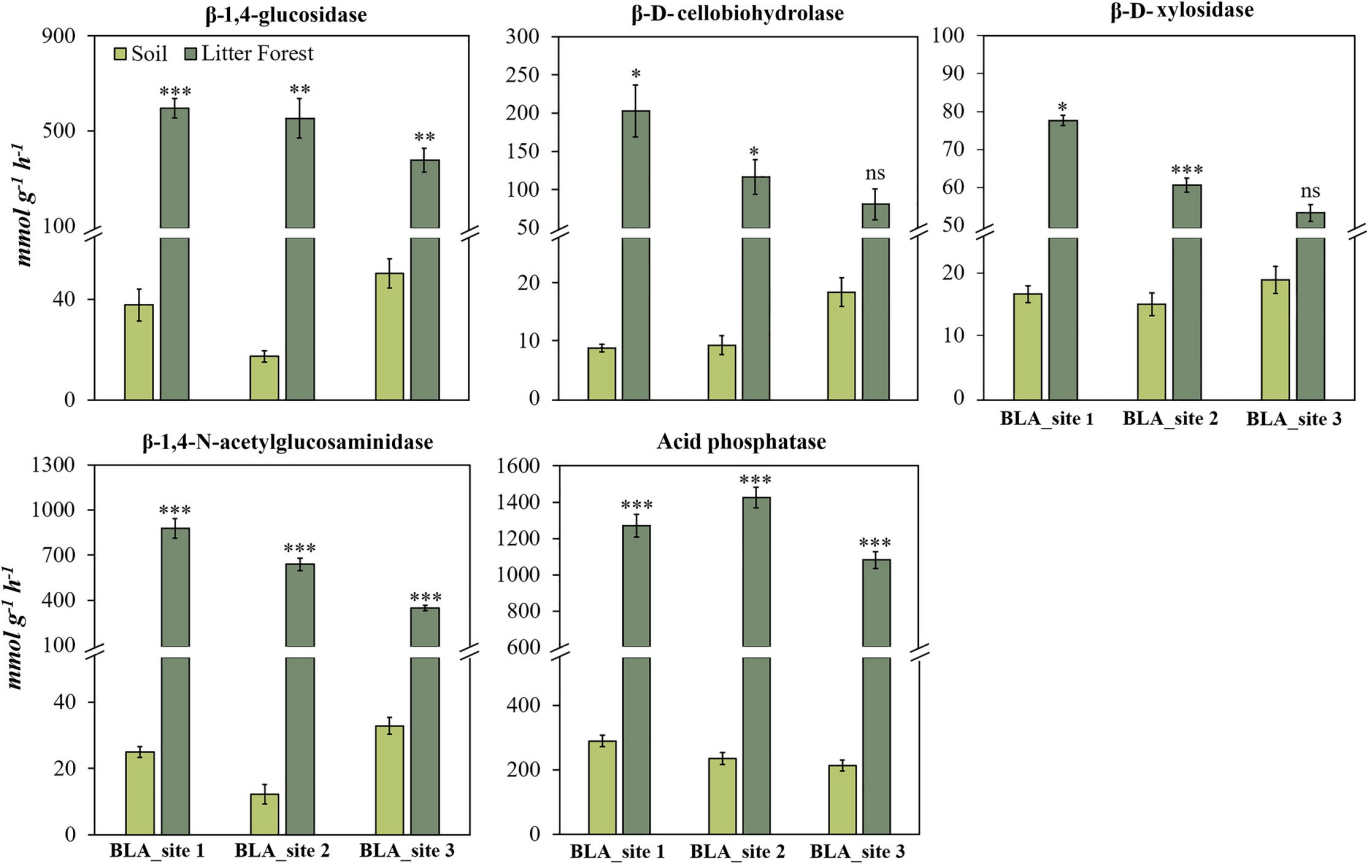
β-1,4-glucosidase, β-D-cellobiohydrolase, β-D-xylosidase, β-1,4-N-acetylglucosaminidase, acid phosphatase enzymes activities in soil and litter of forest mountain landscape sites over an Andes-Amazon transition in Colombian Amazon region. Error bars denote standard error. Asterisks indicate that the means values of soil and litter differ significantly between themselves to the level *p* <0.05 (*), *p* <0.01 (**) and *p* <0.001 (***) for each study site, according to the Student’s Test.

The absolute activity of all five enzymes assessed was higher in the litter than in the topsoil (Figs 2 and 3). The most substantial changes were observed for β-1,4-glucosidase, β-1,4-N-acetylglucosaminidase, exhibiting, for some cases, an activity more than 10 times higher in the litter than in the soil. The activities of β-D-cellobiohydrolase and β-D-xylosidase were higher in the litter than in soil in CTG and SLN in hills landscape sites (Fig 2) and in BLA_site 1 and BLA_site 2 in mountain landscape sites (Fig 3).

### Influence of land use change on the absolute activity of soil enzymes

**Fig 4:**
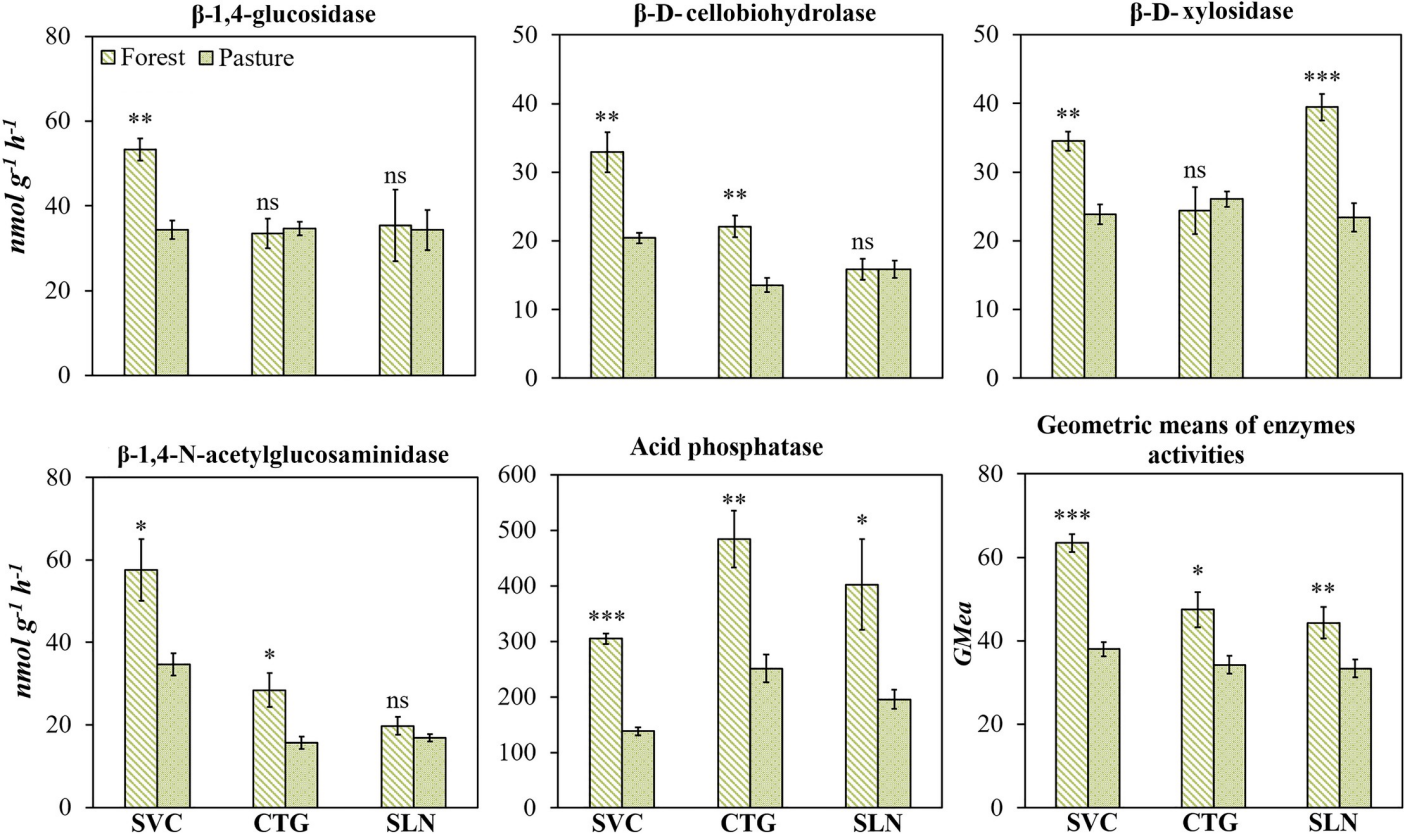
β-1,4-glucosidase, β-D-cellobiohydrolase, β-D-xylosidase, β-1,4-N-acetylglucosaminidase, acid phosphatase enzymes activities in the soil of forest and pastures located in hills landscape (SVC: San Vicente del Caguan, CTG: Cartagena del Chaira, SLN: Solano) of Colombian Amazon region; as well as the index of geometric mean enzyme activity. Error bars denote standard error. The asterisk indicates that the means values of soil and litter differ significantly between themselves to the level p<0.05.

In the hills landscape, the transition from forest to pasture reduced the absolute activity of C, N, and P cycling enzymes, with values, in average, 20% lower in pasture than in forest for β-1,4-glucosidase, β-D-cellobiohydrolase, and β-D-xylosidase, and 33% and 51%, respectively, for β-1,4-N-acetylglucosaminidase and acid phosphatase (Fig 4). Similar trends were noted for enzyme activity based on the *GMea* index (Fig 4).

**Fig 5:**
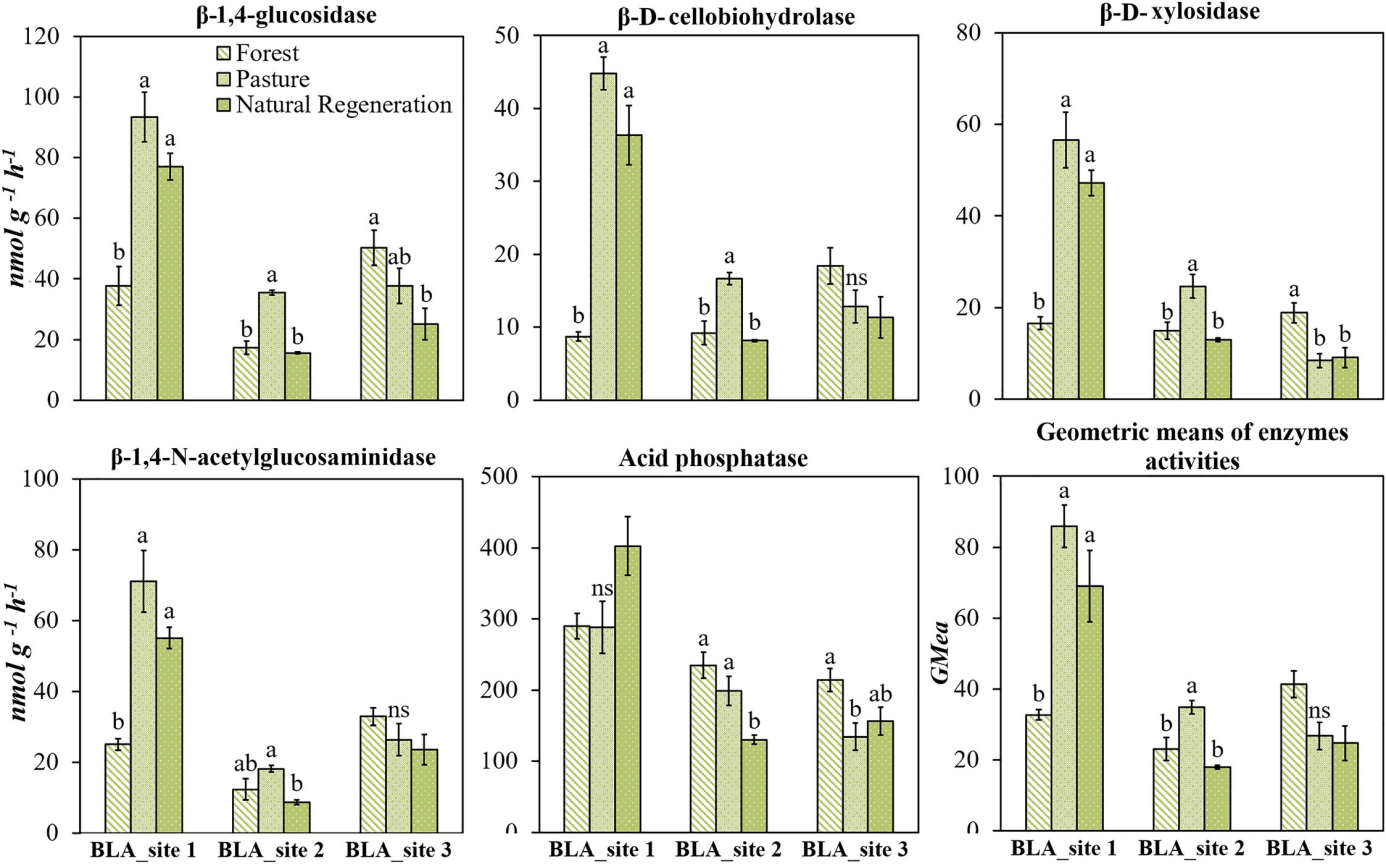
β-1,4-glucosidase, β-D-cellobiohydrolase, β-D-xylosidase, β-1,4-N-acetylglucosaminidase and acid phosphatase enzymes activities, and *GMea* in the soil of forest, pasture and natural regeneration in mountain landscape sites over an Andes-Amazon transition in Colombian Amazon region. Error bars denote standard error. Means followed by the same letters, above the bars, did not differ significantly according to Tukey’s HSD test (*p* <0.05), using “lmer” model.

In the mountain landscape, an increase in the absolute activity of soil C and N cycling enzymes was noted in pasture soil compared to forest soil at BLA_site 1 and BLA_site 2. Abandonment of pastures and regeneration practices caused a decrease in the potential activity of those enzymes in BLA_site 2 (Fig 5). In BLA_site 3, significant differences were detected in β-D-xylosidase and acid phosphatase, with forest showing higher values than pastures.

The *GMea* index indicated an increase in soil enzymatic activity in pasture soil compared to forest soil at BLA_site 1 and BLA_site 2. For the BLA_site 3 no differences were observed among land use types (Fig 5).

### Influence of land use change on soil enzymatic activities weighted by soil C content

**Fig 6:**
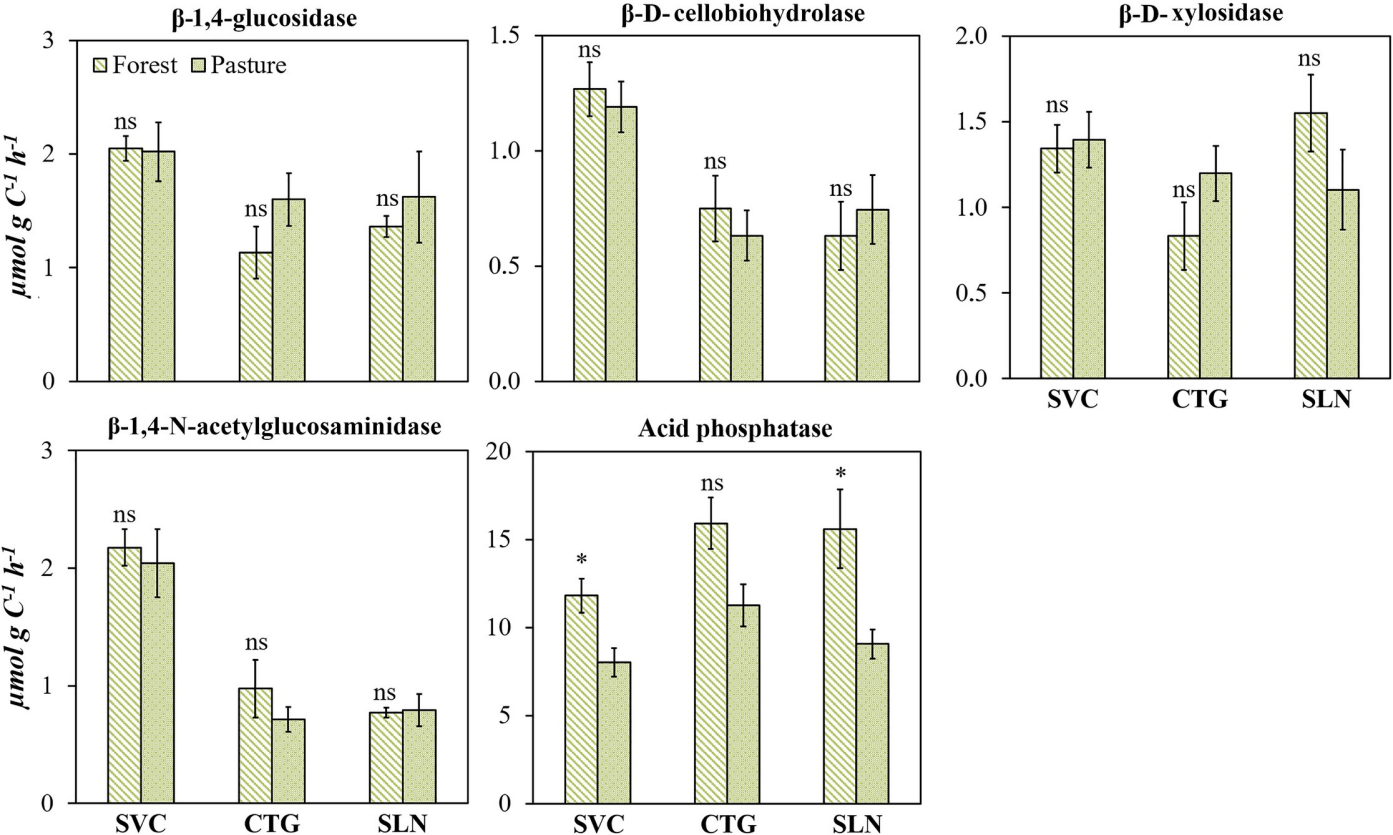
Specific activity per unit of SOC of soil enzymes β-Glucosidase, β-D-Celobiosidase, β-D-Xylosidase, N-acetyl-β-D-Glucosaminidase, acid phosphatase in pristine forest and pastures located in hills landscape (SVC: San Vicente del Caguan, CTG: Cartagena del Chaira, SLN: Solano) of Colombian Amazon region. Error bars denote error deviation. The asterisk indicates that the means values of soil and litter differ significantly between themselves (*p*<0.05) for each study site, according to the Student’s Test.

Soil enzyme activity normalized to per unit of SOC to reveal a relative comparison between the land use type. Under this approach, no changes were observed between forest and pasture soil in hills landscape for the activity of soil enzymes associated with C and N cycling (Fig 6). On the other hand, differences in enzymatic activity of acid phosphatase (*p* <0.05) were verified in all the study areas, indicating a potential decrease (average ~34%) of that enzyme activity with the land use change.

In the mountain landscape, land use change did not alter the activity of β-1,4-glucosidase, β-D-xylosidase, and β-1,4-N-acetylglucosaminidase at BLA_site 1 and BLA_site 3. However, there were significant differences at BLA_site 2, with higher values found in pasture soil than those found in forest and natural regeneration.

**Fig 7:**
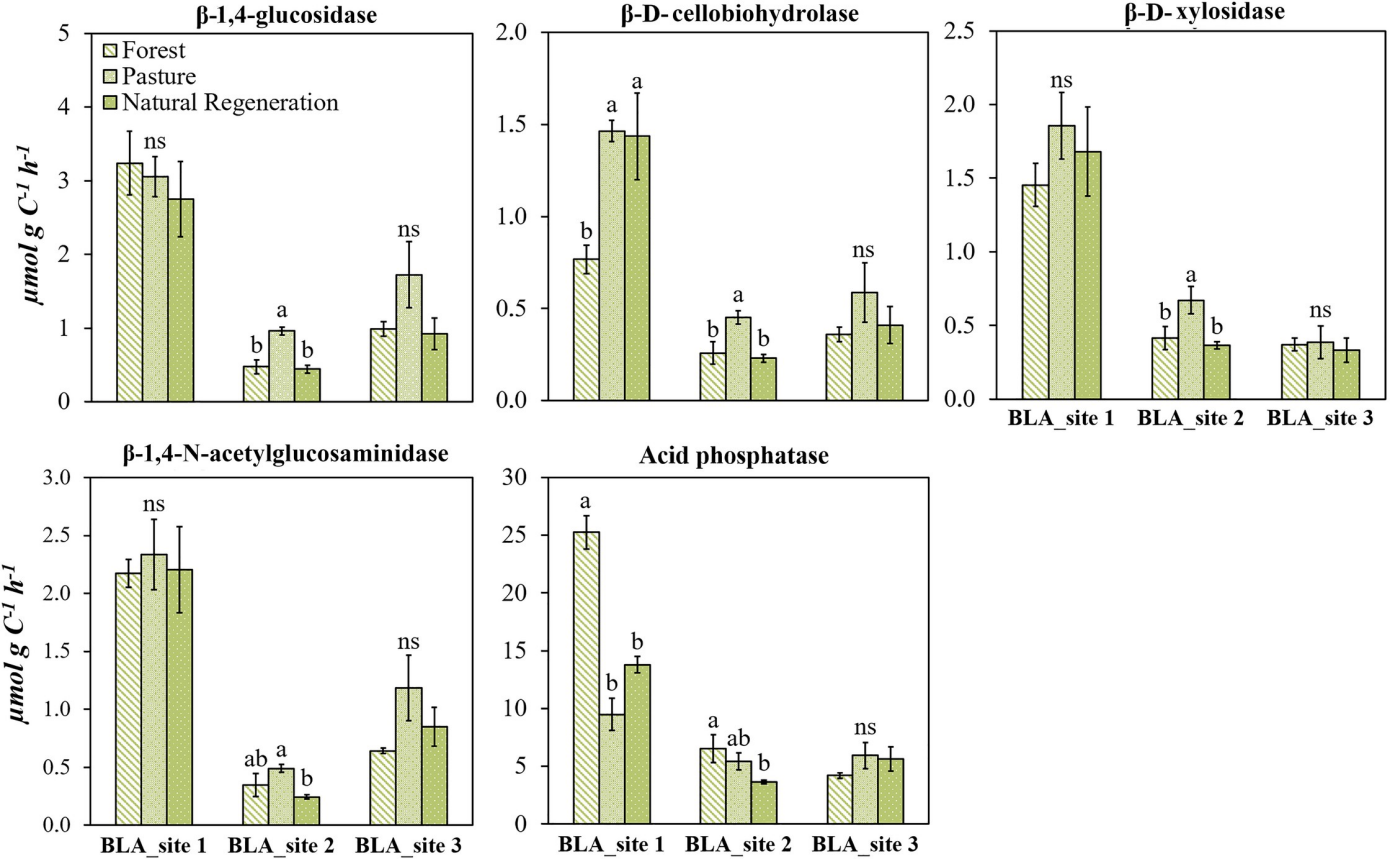
Specific activity per unit of SOC of soil enzymes β-Glucosidase, β-Celobiosidase, β-Xylosidase, N-acetyl-β-Glucosaminidase, and acid phosphatase in the soil of the forest, pasture, and natural regeneration in mountain landscape sites over an Andes-Amazon transition in Colombian Amazon region. Error bars denote error deviation. Means followed by the same lowercase letter in each study site did not differ among themselves according to Tukey’s HSD test (p<0.05).

Changes in the activity of β-D-cellobiohydrolase and acid phosphatase were detected in BLA_site 1 and BLA_site 2, with a higher specific activity of β-D-cellobiohydrolase in pasture than forest, while higher acid phosphatase activity was observed in forest than pastures (Fig 7).

### Stoichiometry enzymes activity

Stoichiometric analysis (relative quantity) of C, N and P enzymes indicated differences in ratios between the soil and litter layer of the forest from all sites (Table 4). Overall, ECN ratios were higher than one in both layers, and lower in the litter layer compared to soil layer. Opposite trends were detected for ECP and ENP ratios, which were generally lower in soil layer compared to litter. Moreover, both ratios were mostly lower than one in all study sites indicating a higher abundance of P-enzymes compared to C and N enzymes (Table 4).

**Table 4:** Stoichiometry enzymes activity in forest litter and soil in Colombian Amazon region.

Landscape	Study site	E_CN_		E_CP_		E_NP_	
		Litter forest	Soil	Litter forest	Soil	Litter forest	Soil
Hill	San Vicente del Caguan	1.03 (0.03) b*	1.19 (0.04) a	0.87 (0.02) a	0.84 (0.01) a	0.86 (0.01) a	0.70 (0.02) b
	Cartagena del Chairá	1.01 (0.01) b	1.33 (0.03) a	0.85 (0.01) a	0.68 (0.03) b	0.84 (0.01) a	0.54 (0.03) b
	Solano	1.08 (0.02) b	1.51 (0.02) a	0.82 (0.01) a	0.75 (0.02) b	0.77 (0.02) a	0.50 (0.01) b
Mountain	BLA_site 1	1.00 (0.004) b	1.29 (0.02) a	0.95 (0.02) a	0.73 (0.01) b	0.95 (0.02) a	0.57 (0.01) b
	BLA_site 2	1.02(0.03) b	1.55 (0.10) a	0.90 (0.02) a	0.68 (0.02) b	0.89 (0.01) a	0.44 (0.04) b
	BLA_site 3	1.06 (0.03 b	1.28 (0.04) a	0.89 (0.02) a	0.83 (0.01) a	0.84 (0.002) a	0.65 (0.01) b

*Means and standard error (SE) followed by the same letter did not differ among themselves according to the T-Student’s test (*p* <0.05).

The transition from forest to pasture and later natural regeneration of pastures did not cause changes in the E_CN_-ratios. However, land use change impacts were observed in the E_CP_ and E_NP_-ratios, as both ratios were generally highest in pasture systems than those in forest (Table 5).

**Table 5:** Stoichiometry enzymes activity in soil under forest and pastures in Colombian Amazon region.

Landscape	Land use	Enzymes		
		C:N	C:P	N:P
Hills		San Vicente del Caguán (SVC)		
	Forest	1.19 (0.04) a*	0.84 (0.01) b	0.70 (0,02) a
	Pasture	1.23 (0.01) a	0.89 (0.01) a	0.72 (0.02) a
		Solano (SLN)		
	Forest	1.50 (0.01) a	0.75 (0.02) b	0.50 (0.01) b
	Pasture	1.52 (0.01) a	0.81 (0.01) a	0.54 (0.01) a
		Cartagena del Chairá (CTG)		
	Forest	1.33 (0.03) b	0.68 (0.03) b	0.51 (0.03) a
	Pasture	1.58 (0.04) a	0.78 (0.01) a	0.50 (0.01) a
Mountain		Belén de los Andaquíes (BLA_site 1)		
	Forest	1.28 (0.02) a	0.73 (0.01) c	0.57 (0.01) c
	Pasture	1.24 (0.03) a	0.93 (0.01) a	0.75 (0.02) a
	Nat. Regeneration	1.24 (0.03) a	0.84 (0.02) b	0.68 (0.02) b
		Belén de los Andaquíes (BLA_site 2)		
	Forest	1.55 (0.10) a	0.68 (0.02) b	0.44 (0.04) b
	Pasture	1.47 (0.03) a	0.80 (0.02) a	0.55 (0.01) a
	Nat. Regeneration	1.68 (0.06) a	0.74 (0.01) ab	0.44 (0.01) b
		Belén de los Andaquíes (BLA_site 3)		
	Forest	1.35 (0.05) a	0.81 (0.01) a	0.60 (0.01) a
	Pasture	1.39 (0.07) a	0.81 (0.03) a	0.58 (0.03) a
	Nat. Regeneration	1.30 (0.04) a	0.74 (0.03) a	0.57 (0.03) a

*Means and standard errors (SE) followed by the same letter in each study site did not differ among themselves according to the Student’s Test in hills landscape and Tukey’s HSD test in mountain landscape (p< 0.05).

## Discussion

### Potential enzyme activities in soil and litter of the tropical forest

Enzymes are essential components within the litter layer of forest ecosystems for catalyzing litter decomposition and nutrient cycling. Our results confirmed higher abundance and activity of C, N and P cycling enzymes in the litter layer and soil, as previously reported in the literature [30, 31]. The microbial production of extracellular enzymes generally is associated with organic C supply and nutrient needs [32], and is primarily stimulated by the plant litter as substrate for microbial growth and activity [33–35].

Several organic compounds of litter may trigger the higher microbial production of enzymes compared to soil. According to the substrate stimulation model, the enzyme activity could be stimulated by the presence of the substrate that it degrades [2]. In this sense, C-degrading enzymes may have been stimulated in the litter when its substrates, e.g., glucosides, disaccharides and cellobioses, increased due to the degradation of cellulose and hemicellulose (Table 2). Furthermore, the glucose, a non-substrate but simple compounds of litter that is readily available and assimilated by microbes, may also cause microbial production of β-1,4-glucosidase [2] by stimulating the microbial growth, which leads to a demand for labile C and hence the production of C-acquiring enzymes [32].

Alternatively, to the substrate stimulation model, soil microbes synthesize many enzymes to facilitate the acquisition of most limiting nutrients in the environment [2]. This could explain the high activity of acid phosphatase and the β-1,4-N-acetylglucosaminidase activity observed in the litter layer at our study sites. An increase in the production of acid phosphatase enzyme was expected since the C:P ratio of the litter was higher than the critical value of 186 (Table 2), above which the conditions are considered to be P-limited for microbial growth and thus, soil microbes generally produce additional P-acquiring enzymes to mineralize organic matter [36].

Regarding the N cycling enzyme, the lower enzymatic acquisition ratios C:N in litter than in soil suggests that in litter the microbial processes were probably limited by N, and thus microbial communities allocated more resources to produce N-acquiring enzymes rather than C.

Overall, the results reported here reflect the importance of litter layer in SOM cycling in tropical rainforest, highlighting its relevance to C sequestration and as a source of nutrients, mainly P and N, to the soil biota and plants. This compartment is usually burned during land preparation for establishing pasture, with long-term implications to restore soil health in those low-fertility soils.

### Changes in SOC, N, and P content and enzyme activities due to transition from forest to pastures

Long-term land use change from forest to pasture within hill landscape led to soil C and N depletion, whereas no significant changes were detected in the mountain landscape.

Those variable soil C and N responses to land transition from forest to pasture corroborate previous studies in the Amazon region [14, 16, 37–41]. In hill landscape the pasturelands are under long-term continuous grazing and poor pasture/soil management practices resulting in reduced C inputs to soil and soil health degradation as previously reported in the Colombian Amazon region [14].

In contrast in less-deep soils such as those studied in the mountain landscape, the shorter period under pasture use and management characterized by a long resting time of grasses have contributed to maintain soil C and N content at similar levels to those observed in native vegetation. Grasses of the genus *Brachiaria* present the ability to add a higher amount of C because of the activity of its root system [42–44]. Thus, site specific land management practices are important factors for impacting SOC storage and must be understood clearly to minimize the losses.

Higher C and N values found in the pasture and natural regeneration areas in BLA_site 1 are likely associated with higher clay content compared to forest soil (Table 1) and not due to land use change. A positive relationship between soil clay and soil C has been pointed out by several studies [45–47].

Regarding P, our study revealed that soil P is limiting in those tropical soils. Overall, low plant-available P contents were also reported by Fernandes *et al*. [48], Soltangheisi *et al*. [49] and Olaya‐Montes *et al*. [14] in Amazon soils. Highly-weathered tropical soils, rich in Fe and Al oxides in clay fraction, can adsorb large amounts of P as insoluble phosphate [50, 51], limiting the availability of inorganic P for plants [48].

Those changes in soil C, N and P induced by the land transition from Amazon forest to pastures seem to be the main driver of soil enzyme activity alterations. It was also reported in tropical forests in Ecuador and Costa Rica by Tischer *et al*. [52] and Cleveland *et al*. [53], respectively. The range of absolute enzyme activity values found in this study are similar to those reported in Brazil and Panama [11, 54–57] but they were lower than those observed in Puerto Rico and Ecuador [52, 58–60], revealing some regional variations even within tropical rainforests.

Overall, the activity of the enzymes studied followed the order acid phosphatase > β-1,4-N-acetylglucosaminidase = β-1,4-glucosidase = β-D-xylosidase = β-D-cellobiohydrolase. The highest acid phosphatase activity levels observed in all the study sites might be a response of plant and microbial communities to the severe P deficiency in these areas (Table 3), as also reported by Soltangheisi *et al*. [49]. Under limited nutrients conditions, the production of enzymes is stimulated by plant roots and microorganisms; therefore, phosphatase could likely be exuded in order to enhance the release of inorganic P from organic P compounds [61], demonstrating in this way the relevance of this enzyme in the mineralization of organic P in tropical Amazon region, as well as the role of soil organic matter in the provision of P for plant growth.

High P degrading enzyme activities have also been reported in both temperate [5, 62] and tropical ecosystems [4, 63, 64]. On the other hand, since chitin is considered the inducer of the activity of β-1,4-N-acetylglucosaminidase, the alterations in the activity of that enzyme can be related to differences in the content of this component (chitin) between soil from forest and pasture areas, more specifically to alterations in macrofauna and fungal biomass, in which chitin is a major structural component [65, 66]. Although we did not measure those variables, previous studies have indicated a reduction in microbial biomass and macrofauna as a response to extensive pastures in tropical regions [67–70].

Compared to global surveys, values of stoichiometry enzymatic activity C:P and N:P lower than 1 observed in all studied soils indicated greater investment toward P acquisition relative to C and N acquisition (Table 4), ratifying the primary microbial P limitation exposed before [36, 71]. Since enzymes control the turnover of soil organic matter by degrading organic molecules to assimilate C, N and P [72], subsequent consequences of organic P-mineralization could lead to further decline of SOC and N [73]. We believe this to be the case in the pastures studied here with chronic soil-P infertility. Thus, pasture management must consider increasing crop diversity to enhance mycorrhizal interactions, which are capable of acquiring inorganic soil-P and contribute to meet the P needs of plants.

Ratios of C:N acquiring enzymes were greater than one, which indicated that microbes allocated more resources to produce C-acquiring enzymes rather than β-1,4-N-acetylglucosaminidase. Although those results could suggest that a C-limiting condition is present in all the areas, preliminary studies pointed out that enzymes degrading SOM to provide energy (C) versus nutrients (N and P) may be regulated by resource availability [1], with C-degrading enzymes being stimulated by elevated concentrations of their substrates and end-products [2]. Furthermore, since some microbes can use labile C to decompose recalcitrant organic matter to obtain N (N-mining theory) the microbial N-acquisition can be alleviated by C-acquisition [74], which is consistent with a soil C:N ratio in all sites below the threshold of 14.3 [75].

As with individual enzyme activity per unit of soil mass, the *GMea* index was altered with land use change (Figs 4 and 5) in all the study sites, proving to be a suitable indicator for estimating the soil microbial community response due to pasture establishment. To decouple the changes in soil enzyme activities from SOC changes, we calculated weighted enzymatic activity based on SOC base. In contrast to the absolute enzyme activities, although land use transition from forest to pastures decreases the SOC and those enzyme activities when considered individually in hills landscape, enzyme activities as a proportion of SOC are maintained. Therefore, after a long-term pasture establishment, the rate of reduction in SOC is greater than enzyme activity, thereby maintaining high metabolic activity likely promoted by N and P limitations.

This pattern was also found in mountain landscape, with increases in the activity of β-1,4-glucosidase, β-D-cellobiohydrolase and β-D-xylosidase in pastures of BLA_site 2, which could also provide insight into how suitable is the SOM for degradation by C and N cycling enzymes in pasture areas, thus providing a measure of organic matter quality [71]. Both the turnover of roots and root exudation are important sources of SOM in pastures, supplying a variety of labile carbon compounds to soil [76]. Experiments studying the effect of litter and root plants on soil enzyme activity have reported an increase in C and N-degrading enzyme concentrations when root growth is allowed due to active transport of labile carbon from roots to associated microbial communities [77, 78].

On the other hand, the phosphatase activity per unit of carbon followed the same trend observed in absolute activity, with the soil C losses in the hills landscape being accompanied by a decrease in the abundance of P-acquiring enzyme per unit of C. Moreover, non-changes in SOC in mountain landscape were associated with the absence of alterations in the specific activity of acid phosphatase. This could be explained by the decrease of soil N due to pasture establishment, which may have a determinant role in regulating acid phosphatase activity according to the results obtained by [79], who analyzed measurements of phosphatase in natural soils around the world. Overall, the development of a soil microbial community in pastures with enhanced catabolic activity and greater biomass turnover could have led to the synthesis of C and N cycling enzymes comparable to the forest and natural regeneration sites. Our results also support the hypothesis that enhanced soil organic matter quality, specifically the presence of substrates, may trigger microbial production of studied enzymes related to C, N and P cycles.

## Conclusions

The litter layer plays a key role in the nutrient budget of the Amazon rainforest. By harboring a considerable pool of extracellular enzymes responsible for regulating its decomposition, this compartment becomes an essential component for C sequestration and the biogeochemical cycling of elements in that ecosystem. With the land transition from forest to pastures, the litter layer is lost. Then, the direction and magnitude of changes in soil enzymatic activities depended largely on the management of pastures, with SOC and N losses and reduced absolute activity of soil enzymes induced by long-term pastures under continuous grazing (25 years). Values of stoichiometry enzymatic activity indicated a primary microbial P limitation in the study areas, which could lead to an increased use of soil C by microbial metabolism in the search for P causing a further decline of SOC and N in pastures.

With the land use transition from forest to pastures a soil microbial community with high catabolic activity per C unit is developed, which could affect SOM cycling with implications in soil health and the provision of soil-related ecosystem services.

Overall, this study provides the first regional data on soil enzymatic activity in tropical forests and pastures of the Colombian Amazon region, providing new insights to a better understanding of land use change effects on the soil biological activity and the dynamic of biogeochemical cycles.
